# Cognitive Load Estimation in VR Flight Simulator

**DOI:** 10.16910/jemr.15.3.11

**Published:** 2023-07-05

**Authors:** P Archana Hebbar, Sanjana Vinod, Aumkar Kishore Shah, Abhay A Pashilkar, Pradipta Biswas

**Affiliations:** CSIR-National Aerospace Laboratories Bengaluru, Karnataka, India; Indian Institute of Science (IISc), Bengaluru, Karnataka, India; All India Institute of Medical Sciences, New Delhi, India

**Keywords:** Human factors, virtual reality, cognitive load, ocular parameters, eye gaze, flight simulator, EEG, task engagement

## Abstract

This paper discusses the design and development of a low-cost virtual reality (VR) based flight simulator with cognitive load estimation feature using ocular and EEG signals. Focus is on exploring methods to evaluate pilot’s interactions with aircraft by means of quantifying pilot’s perceived cognitive load under different task scenarios. Realistic target tracking and context of the battlefield is designed in VR. Head mounted eye gaze tracker and EEG headset are used for acquiring pupil diameter, gaze fixation, gaze direction and EEG theta, alpha, and beta band power data in real time. We developed an AI agent model in VR and created scenarios of interactions with the piloted aircraft. To estimate the pilot’s cognitive load, we used low-frequency pupil diameter variations, fixation rate, gaze distribution pattern, EEG signal-based task load index and EEG task engagement index. We compared the physiological measures of workload with the standard user’s inceptor control-based workload metrics. Results of the piloted simulation study indicate that the metrics discussed in the paper have strong association with pilot’s perceived task difficulty.

## Introduction

Modern-day aviation involves incredibly sophisticated technologies. Recent research on cockpit design of combat aircraft, often under the umbrella term of 6^th^ generation cockpit design, is investigating novel modalities of interactions. Adaptive pilot vehicle interfaces (PVI) and wearable cockpit features are being studied (39; 40). New modalities of interaction like brain-computer interface or eye gaze-controlled systems present new challenges and opportunities for PVI inside cockpit. Any such new PVI design evaluation necessitates human engineering methods to understand the variations in the cognitive load experienced by the users. Many researchers have studied different means of measuring cognitive load (2; 6; 33; 50). Cognitive load may be quantified by subjective, physiological and performance-based measures. User’s assessment of the system is captured through questionnaires in the subjective measure. Performance based methods quantify the same by capturing how well he/she performs a given task. Physiological methods like pupil dilations, EEG signal variations, heart rate variability, galvanic skin responses and so on measure user’s physiological state. All these methods have their own drawbacks for standalone implementation. For example, though subjective measures are simple to administer, their accuracy depends on user’s prior knowledge and bias. Performance based methods are very task specific. Advantage with the physiological methods is that it enables continuous monitoring of the workload. However, they are not reliable in cases wherein change in physiological indicators may be due to factors not related to workload. They do not explain the cause of the variations. Hence, due to the multi-dimensional characteristic of cognitive load, a combination of the above methods needs to be used for estimating cognitive load. In this study, we used physiological measures, namely ocular and EEG parameters, along with pilot workload using inceptor time histories to estimate pilot’s cognitive load.

Furthermore, design evaluations of any new aircraft technology need to be carried out in a realistic cockpit environment. Hence, an aircraft flight simulator plays a significant role in the development and testing of such new technologies. Any aircraft program encompasses of different flight simulators throughout its design, deployment and maintenance life cycle. The fidelity and complexity of a simulator depends on the application requirement (9). As more hardware interfaces get added to the simulator, the cost to build and maintain the simulator goes up. Virtual reality based flight simulator is a promising low cost and modular modality to offer a more immersive and adaptable experience for applications wherein lot of design iterations are involved. Augmented, virtual and mixed reality-based cockpits are already being used for cockpit/cabin design evaluations in terms of reachability and visibility, for providing inspection and maintenance training to engineers (46) and are considered even for real time deployment (14). Oberhauser et al., (32) compared VR based flight simulator with a conventional hardware simulator. Authors found that ability for rapid prototyping in VR based simulators makes it a viable tool during the early phases of design process. VR based simulators are hence proven to aide human-in-the-loop testing of new systems and their interactions with pilot.

In this paper, we discuss the development of a virtual reality-based flight simulator with automatic cognitive load estimation feature. The main aim of this study is to evaluate different methods for estimating cognitive load in a VR environment. The simulator exploits the existing tools such as Unity SDK engine and has generic USB based hardware. We employ HTC Vive Pro Eye as the VR head-mounted display with an in-built eye-tracker, Emotiv 32 electrode EEG headset and a Thrustmaster HOTAS to control the aircraft.

Next, we developed cognitive load estimation algorithms based on ocular and EEG signals. Ocular parameters are based on pupil dilation dynamics, gaze fixations and gaze distribution. EEG signal-based measures are for estimating task load and task engagement. Later, we developed an AI agent model that interacts with pilots. Selection of this scenario is the fact that old F-16 fighters are being converted to unmanned targets (QF-16) by Boeing for US Airforce pilot training (7). QF-16 is remotely piloted aircraft that helps pilots to practice air-to-air combat skills. In this study, we consider an AI enabled target aircraft. Aim here is to generate different one-on-one air combat scenarios through an AI agent and thereby evaluate effect of pilot-aircraft interactions on pilot’s cognitive load. We conducted a user study with twelve Airforce test pilots for five AIAgent configurations. Results indicate that the developed algorithms estimate cognitive load accurately in the VR environment.

Rest of the paper is organized as follows. Next section presents the simulator framework. Section 3 describes conduct of the user study and analysis, followed by results and discussions in section 4. Concluding remarks are addressed in section 5.

## VR Flight simulator framework

**Figure 1.**
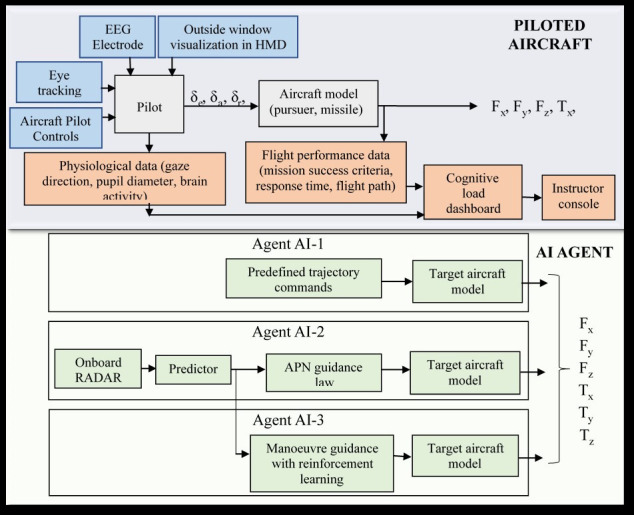
VR flight simulator framework

Figure 1 shows the overall framework of the VR flight simulator. The aircraft that the pilot/engineer operates is known as the piloted aircraft (PA). The autonomous aircraft is termed as the AI Agent. AI agent acts as the enemy aircraft in the scenarios considered for the study reported in the paper. Pilot has to track the AI agent and fire missile when commanded on the VR headset.

Simulator uses Unity engine, which is one of the widely used game development platform due to its rapid prototyping capability and compatibility with VR displays and the interaction tools (www.unity3d.com).

PA is modelled to mimic F-16 aerodynamics. ‘Aircraftphysics’ asset provided by Unity is used to apply aerodynamic forces and torques to the rigid body aircraft. The moments acting on the aircraft is the sum of impact of individual control surfaces.

**Figure 2.**
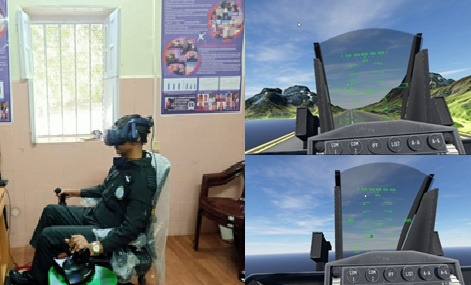
VR flight Simulator: hardware Setup (left), visual rendering on HMD on ground (right top) and during target tracking (right bottom)

A generic head-up-display symbology and other controls such as flaps, landing gear, airbrakes, toe brakes and parking brake functionalities are modelled in the system (Figure 2). We have used physiological measurement methods such as EEG signals and ocular parameters for estimating participant’s cognitive load. Gaze vector is also used for target pointing and selection. Pilot controls such as throttle, rudder pedals and the pilot control stick are from USB based Thrustmaster HOTAS.

A generic missile model is developed for the simulator. User can release the missile with the fire button on the pilot control. Impact of missile hit is dependent on the range between the missile origin to the impact point, with a predefined radius.

We have used HTC Vive Pro Eye HMD for rendering cockpit view and outside window scenery to the participants. The HMD gives a diagonal FOV of 110° and a resolution of 1440 X 1600 pixels. HTC Vive Pro Eye has an inbuilt eye tracker which is used to record ocular parameters such as x/y gaze direction vectors, left/right pupil positions and pupil size. All required data is synchronized with the aircraft data and is acquired at 120Hz. We used HTC Vive’s SRanipal SDK (13 and 27) along with Tobii’s XR SDK version 1.8.0 (45) for eye tracker data recording. Tobii XR SDK uses Gaze-to-Object Mapping (G2OM) algorithm to determine what the user is looking at. We recorded gaze direction from Tobii XR and pupil diameter from SRanipal SDK. We also used Emotiv 32 channel EEG headset to record brain activity.

AI agent is modelled as a point mass model. It has an onboard tracking radar that detects targets within the coverage of 1.2 km. The radar scans the targets, processes the data and estimates the target position. RADAR acquisition and processing latency of 400ms is added to the output of radar data. This latency accounts for the time lag between receipt of a signal or data and its appearance on the pilot display and the propagation delay (22).

### AI agent’s evasive maneuvers

A simple pursuit-evade combat strategy is implemented in this study. User controlled aircraft acts as the pursuer (P) and the AI agent is the evader (E). User gets an indication to launch the missile once the AI agent is within the chase range of the missile. $V_{p}$ is the PA velocity that is controlled through user’s throttle control. Evader initially starts at a constant velocity. Start position of the AI agent is randomized to simulate the real-life behavior. AI agent is implemented through three different approaches as described in the subsequent sections.

### AI agent 1: No guidance

**Figure 3.**
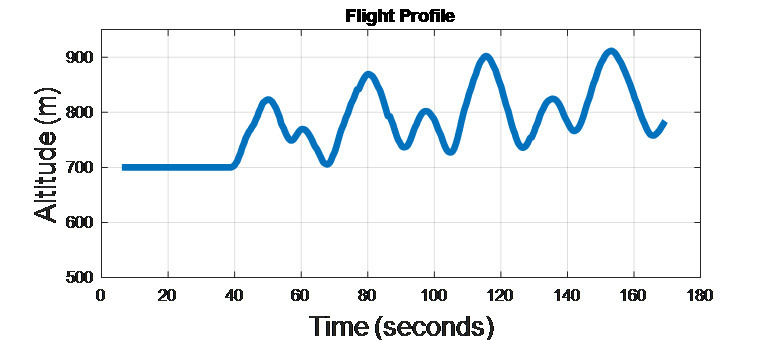
AI agent 1 Flight profile

Evader moves with a constant forward velocity, unaware of pursuer’s actions. His/her flight path has a constantly varying altitude as shown in the Figure 3.

### Agent 2: Augmented Proportional Navigation guidance (APN)

**Figure 4.**
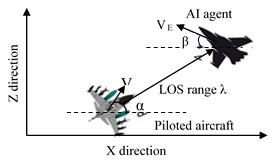
Two-dimensional scenario

In this scenario, we considered a simplified one pursuer (PA), one evader (AI agent) model shown in Figure 4. Here, Y axis denotes the altitude, Z is the forward range and X denotes the lateral movement. The subscripts e and p represent evader and pursuer respectively. The approach taken is that if evader remains within the chase range or on the line joining the evader to pursuer (defined by the line-of-sight angle), it will eventually hit the target. APN algorithm computes acceleration command to steer the aircraft heading (26). The computed acceleration is proportional to the line of sight (LOS) rate ( $\dot{\lambda }$) and the closing velocity (Vc).

LOS distance λ between pursuer and evader is given in Equation 1. λ is defined as the Euclidian distance between the two.

Equation 1 $$\lambda =\;\sqrt{{\left(x_{e}-x_{p}\right)}^{2}+{\left(y_{e}-y_{p}\right)}^{2}+{\left(z_{e}-z_{p}\right)}^{2}}$$

Closing velocity $V_{c}$ between the evader and the pursuer is $V_{c}\;=\;V_{p}\;–\;V_{e}$

Normal acceleration command is given in Equation 2 (26)

Equation 2 $$a_{N}=N\;V_{c}\dot{\lambda }\;+\;a_{Nt}N/2$$

Here, N is the navigation constant, taken as 3 and $a_{Nt}$ is the target acceleration.

Objective of evader is to maximize the distance between evader and pursuer so that evader’s survivability increases. Evader’s velocity is made proportional to the line-of-sight rate such that $V_{e}>\;V_{p}$.

### Agent 3: AI Agent trained using reinforcement learning algorithm

Reinforcement learning (RL) based guidance strategy (25) is developed for training the third AI agent. We used Unity’s machine learning (ML)-Agents Toolkit to develop and train the RL agent.

Unity scene behaves as the environment and a Python API is used for training. Agent’s goal is to maximize the cumulative rewards. Rewards were given if the following conditions were satisfied (47) -

1. Distance between PA and AI agent dt: dmin<dt<dmax. This condition ensures that evader is within the attack range.
2. Deviation angle in degrees μ< μmax.
3. Aspect angle in degrees η< ηmax

The corresponding equations are given in Equation 3.

Equation 3 $$dt=\sqrt{{\left(x_{p}-x_{E}\right)}^{2}+{\left(y_{p}-y_{E}\right)}^{2}+{\left(z_{p}-z_{E}\right)}^{2}}$$

$$\mu =180+\psi_{p}-\tan^{-1}\left(\frac{\left(x_{p}\;-\;x_{E}\right)}{\left(z_{p}-z_{E}\right)}\right)*\frac{180}{\pi }$$

$$\text{η}=\psi_{E}-\tan^{-1}\left(\frac{\left(x_{E}\;-x_{p}\right)}{\left(z_{E}\;-z_{p}\right)}\right)*\frac{180}{\pi }$$

Reward function consists of following advantage positions:

- Distance from pursuer is more than the chase range of the missile.
- Angle between the line-of-sight vector and the aircraft heading is larger than the maximum predefined angle.

- Aspect angle, which is the angle between pursuer’s longitudinal axis and the line joining from pursuer’s tail to agent’s nose, is larger than the predefined angle.

## User Study: Study of Pilot’s interactions with aircraft

### Mission preparation

**Figure 5.**
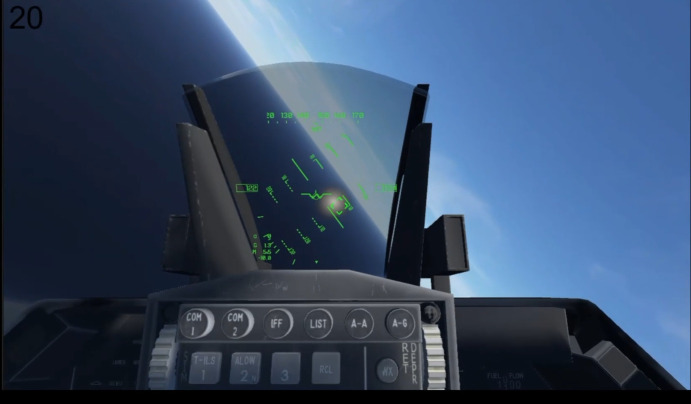
Missile Fire

The test scenarios in the study is to simulate dogfight battle scenarios with one AI agent in VR. AI agent’s initial position is set randomly at the start of the simulation. Simulation starts with the piloted aircraft on the runway and the AI agent at an initial altitude of 700 m. Pilot has to track the AI agent and fire the missile when commanded on the VR headset (Figure 5). To increase the missile fire accuracy, only six missiles are made available during each simulation. Simulation is terminated either when AI agent is shot down or when there is a ‘Timeout’ message displayed on the VR headset.

We repeated the simulations with three AI agents mentioned in the previous section. Table 1 gives details on design and conduct of the test scenarios. Pilot’s control strategy-based PIW metric is taken as the baseline. Physiological parameters estimating participant’s cognitive load are the dependent variables used in the analysis.

**Table 1:** Test scenario

Task	Details
C1	Give throttle input to increase speed. Take off when speed in > 125 m/s. Fly wings level while maintaining speed of < 130 m/s. AI agent’s initial altitude is 700m and shall maneuver as per AI agent 1.
C2	Give throttle input to increase speed. Take off when speed in > 125 m/s. Fly wings level while maintaining speed of < 130 m/s. AI agent’s initial altitude is 700m and shall maneuver as per AI agent 2.
C3	C2 with a degraded radar latency of 800ms.
C4	Give throttle input to increase speed. Take off when speed in > 125 m/s. Fly wings level while maintaining speed of < 130 m/s. AI agent’s initial altitude is 700m and shall maneuver as per AI agent 3.
C5	C4 with a degraded radar latency of 800ms.

### Participants

We conducted a user study with twelve Airforce test pilots; each for 5 test conditions. These professionally qualified test pilots are trained for quick decision making in difficult situations. By definition, test pilot is a pilot who is specially trained for evaluating yet to be certified aircrafts (43). Pilots participating in the study have an average age of 40 years and a flying experience of over 3500 hours. None of the pilots were wearing any prescription lenses.

### Procedure

All simulations were carried out with the same hardware and in same environmental conditions. Participants were first briefed about the task to be carried out and were given ~15 minutes of flying time to get accustomed to the simulator set up. EEG electrodes were soaked in saline water for a minimum of 30 minutes before start of simulation for each participant. We carried out five simulations for each participant. Test scenarios were carried out randomly to nullify the order effect. Experiments for each participant started with EEG headset calibration and Vive Pro eye tracker 5-point calibration. Interpupillary distance, which is participant specific, was adjusted for better viewing experience and to reduce the eye strain. Participants were asked to be in relaxed state for the first 5 seconds before start of each simulation and this data is recorded as the start condition for all the parameters.

### Data Processing and analysis

This section discusses the procedure employed to analyse the ocular, EEG and flight parameter data. Different metrics formulated for analysing the participant behaviour and estimating the experienced cognitive load are also described.

### Ocular parameters

- Pupil dilation dynamics

Literature review reveals that pupil diameter (PD) increases with increase in exerted mental effort (35; 41). Like other biological signals, pupil dilation is a non-stationary signal (24). Hence rather than using Fourier transform, which give only frequency resolution, we have used wavelet transform that gives both time and frequency resolution. Pedrotti, et al. (34) also proposed wavelet analysis to extract relevant low frequency PD signal features by discarding the high frequency noise.

We analysed timeseries PD data through multi resolution analysis (MRA) to compute PD fluctuations. Data frames in which eye measurements are marked as invalid by SRanipal are removed before analysis. This may either be due to failures to detect pupil (because of loss of tracking) or due to eye blink. Further, data is interpolated using cubic spline interpolation to fill in the missing timestamps.

PD data is then normalized based on the maximum value of first 5 second reference data as given in Equation 4.

Equation 4 $$\%PD=\frac{\left({PD}_{raw}\left(t\right)-{PD}_{baseline}\right)}{{PD}_{baseline}}*100$$

Where ${PD}_{raw}\left(t\right)$ ${PD}_{raw}\left(t\right)$is the pupil diameter at time t. This correction shall remove the inter-participant variability.

**Figure 6.**
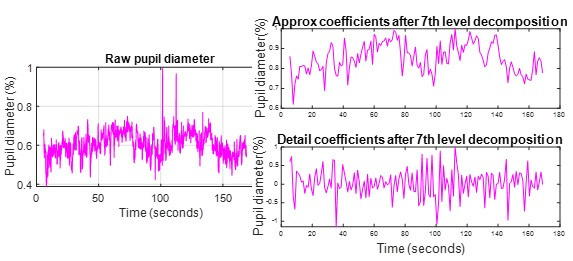
DWT Coefficients: Normalized PD signal (left), After 7th level decomposition (right).

Later, we de-noised the PD signal using MRA to reproduce the low frequency component of the signal. We have used 1D-DWT with 7 level decompositions. Haar wavelet is chosen as the mother wavelet (34). Each wavelet decomposition level has a down sampling by a factor of 2. The initial sampling rate is 120Hz. Figure 6 shows the normalized pupil diameter along with its approximation and detail coefficients after 7th level decomposition.

Standard deviation ( StdDevPD ${StdDev}_{PD}$) of 7th level approximation data is computed as an indicative of the low frequency PD variations with cognitive load (Equation 5).

Equation 5 $$StdDevPD=\;\sqrt{\frac{1}{N-1}\sum_{i=1}^{N}{\left({PD}_{i}-{PD}_{mean}\right)}^{2}}$$

Where ${PD}_{mean}=\frac{1}{N}\sum_{i=1}^{N}{PD}_{i}$

N is the total number of data points and ${PD}_{i}$ ${PD}_{i}$ is the ith data point of the processed pupil diameter discussed earlier in this section.

b. Gaze fixation analysis

Fixation is a type of eye movement which indicates that visual information is registered by the brain. Saccades are rapid eye movements between one fixation to another. Fixation has been used to make inferences on level of attention and cognitive processing of a person (31 and 42). Fixation can be determined either by computing fixation frequency or duration on the area of interest.

We detected fixations and saccades from gaze direction data through velocity threshold using fixation identification method (1; 30). Firstly, we computed angle between consecutive gaze direction vectors. We then calculated angular velocity as change in angle divided by the time increment. We identified fixation based on a velocity threshold of 30 degree/sec. If the angular velocity is lesser than the velocity threshold, it is treated as a fixation. Successive fixations are computed as one fixation. Subsequently, we computed fixation rate as the ratio of total number of fixations to total task duration. Mean fixation duration is computed as the ratio of sum of all fixation durations to total number of fixations.

Liu et al. (27) has reported several studies with contradicting result patterns for fixation parameters. Authors argue that fixation rate increases in conditions where number of items/information to be processed for decision making is higher than that can be accommodated within a single fixation. Contrary to this, fixation rate reduces in situations which demand greater imagination and manipulations, wherein all items related to the task are processed within a single fixation. Hence variations in fixation rate is highly dependent on the nature of task under study.

c. Gaze distribution analysis

Spatial gaze distribution patterns are sensitive to variations in cognitive load. In general, low workload is associated with more deterministic and repetitive visual scanning pattern (5). Di Nocera et al. (12) proposed nearest neighborhood index (NNI) to analyze distribution of gaze position. NNI is the ratio of mean of nearest neighbor distance to mean random distance. Nearest neighbor distance is the distance between each gaze point to the next gaze point near it. A higher value of NNI denotes that visual scanning is more randomly distributed in space. Hebbar et al., (21) gives details on the implementation of NNI.

### EEG parameters

We used Emotiv EPOC Flex-32 channel saline sensor based wireless EEG headset to measure brain’s electrical activity. Raw EEG data for each electrode (in µV) is captured at 128Hz. Substantial amount of signal processing and filtering is carried out within the Flex headset to remove the ambient noise and harmonic frequencies (48). Flex headset has in-built data pre-processing algorithms which includes a high-pass filter of 0.2 Hz, a low-pass filter of 45 Hz, a notch filter at 50 and 60 Hz, digitization at 1024 Hz and further filtering using a digital 5^th^ order sinc filter. The data is further down sampled to 128 Hz for transmission.

Sensor data is processed into four frequency bands: theta (4-8Hz), alpha (8-12 Hz), beta (16-25Hz) and gamma (25-45 Hz). Emotiv also provides average band power (in µV^2^/Hz) for each frequency band computed using fast fourier transform (FFT). Before applying FFT, the data is processed through a hanning window of size 256 samples that is slid by 16 samples in each iteration to create a new window (15; 20). Both the raw sensor data and the average power per frequency band for each sensor is stored for each simulation.

Data from two participants could not be used due to poor contact quality while recording. Metrics used for EEG analysis are discussed below.

d. EEG Task load index (TLI)

EEG based TLI is the ratio of anterior frontal and frontal midline theta energy to mean parietal alpha energy (17; 23). Studies ( 49; 8) reveal that theta power increases with increasing tasks that demands sustained concentration. TLI is a proven metric that increases with cognitive tasks such as problem solving, integration of information and analytical reasoning (4).

**Figure 7.**
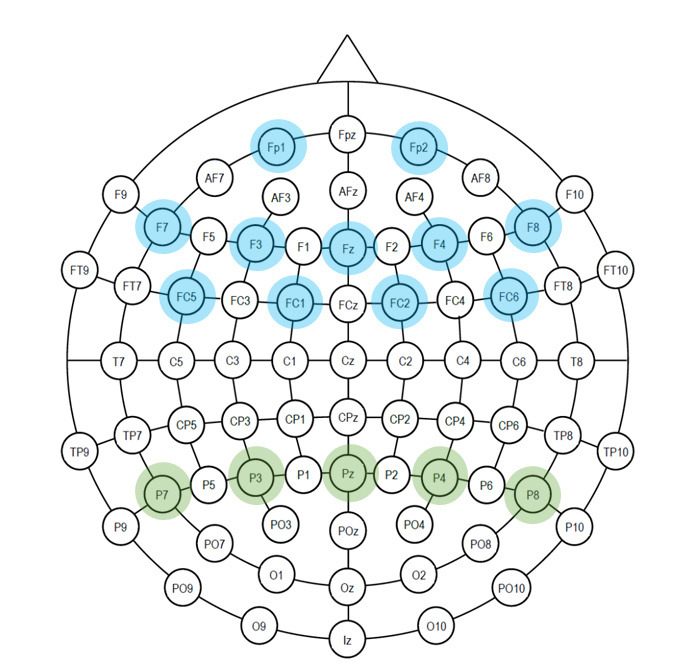
Emotiv EPOC Flex- Electrodes placement: Electrodes used for TLI (Fz, Fp1, Fp2, F3, F4, F7, F8, FC1, FC2, FC5, FC6 electrodes - for theta frequency band; P7,P3,Pz,P4,P8 for alpha band)

The electrode positions are shown in Figure 7. We have computed TLI as ratio of power of theta frequency band of Fz, Fp1, Fp2, F3, F4, F7, F8, FC1, FC2, FC5, FC6 electrodes to power of alpha frequency band of P7, P3, Pz, P4, P8 electrodes. Figure 7 shows the default electrode positions, while highlighting the electrodes used for computing TLI.

e. Task engagement index

Freeman et al., (16) derived an EEG signal-based Engagement index to explore user’s engagement, alertness and vigilance to a task conducted to evaluate an adaptive automation system. Prinzel et al., (37) also recommended TEI for designing automated systems. Pope et al., (36) developed a biocybernetics system using TEI to evaluate flight deck automation based on operator’s engagement in the task.

EEG task engagement index (TEI) is defined as the ratio of beta band power to sum of theta and alpha band power (beta power/(alpha power + theta power)). An array of different montages has been used in literature for TEI measurement. We evaluated five different montages – F4, F3, F7, F8 (16); Pz, P3, P4, Cz (36); Pz, P3, Fz, C3 ( 10); Fp1 (44) and Pz, P4, Fz, C4 (11). We analyzed all the five montages and observed that F4, F3, F7 and F8 electrode combinations exhibited higher correlation. We thus discuss this montage in this section.

**Figure 8.**
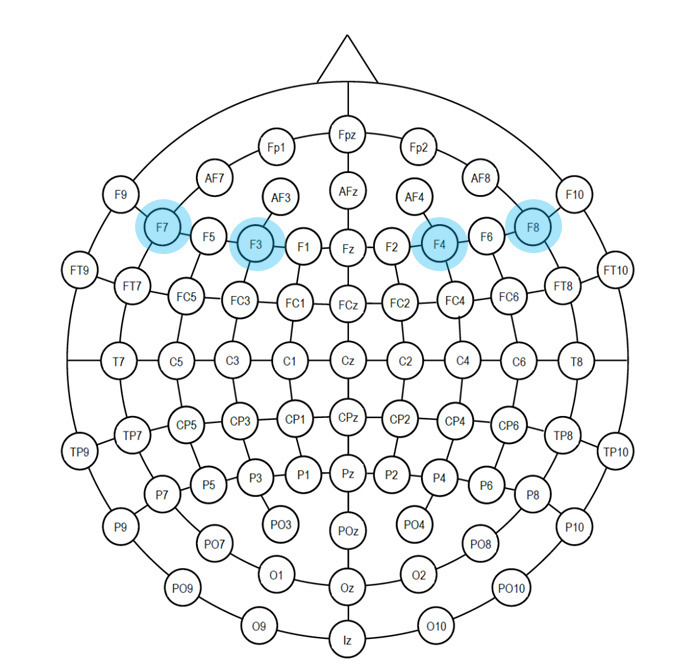
Emotiv EPOC Flex- Electrode placement: Electrodes used for TEI (F4, F3, F7 , F8 – for theta, alpha and beta frequency bands)

Average beta, alpha and theta bands of F4, F3, F7 and F8 electrodes (Figure 8) are considered to compute TEI. TEI reflects information gathering, visual processing, and allocation of attention as it tracks the demand for sensory processing and attentional resources.

### Pilot control parameters

Task difficulty and time pressure have a direct relationship with cognitive load changes (18). Time history of pilot control inputs provide inference on task difficulty. We used two standard user’s inceptor control-based metrics as described by Hanson et al. (19) for the analysis:

- Duty cycle (DC): DC denotes the total percentage of time participant uses his/her controls. DC increases as the task demands higher control as given in Equation 6.

Equation 6 $$Duty\;Cycle=\;100\%*\frac{1}{t_{n}-\;t_{2}}\sum_{i=2}^{n}x_{i}$$

Where $x_{i}=\;\left\{ \begin{array}{c} 0\;\;for\;\frac{\delta_{i}-\;\delta_{i-1}}{t_{i}-t_{i-1}}<noise\;threshold\;and\;\left|\delta_{i}\right|<\delta_{\max}\;\\ 1\;\;\;\;\;otherwise\;\;\;\;\;\;\;\;\;\;\;\;\;\;\;\;\;\;\;\;\;\;\;\;\;\;\;\;\;\;\;\;\;\;\;\;\;\;\;\;\;\;\;\;\;\;\;\;\;\;\;\;\;\;\;\;\;\;\;\;\;\;\;\;\;\;\;\; \end{array}\right.\;$

$t_{2}$ is the start time +1 and $t_{n}$ is the end time of the data set; n is the number of data points; $\delta_{i}$ are the discrete values of the stick deflections in degrees and $\delta_{\max}$ is the maximum stick deflection.

Noise threshold is taken as 0.5% of inceptor’s total displacement range per time increment.

- Aggressiveness: Aggressiveness describes how rapid are the control inputs. Aggressiveness is measured in terms of rate of change of pilot stick inputs (Equation 7). Increase in aggressiveness correlates with more random and abrupt control inputs; which is in turn related to higher task demands.

Equation 7 $$Aggressiveness=\;\sqrt{\frac{1}{n-1}\sum_{i=2}^{n}{\left(\frac{\delta_{i}-\;\delta_{i-1}}{t_{i}-t_{i-1}}\right)}^{2}}$$

Computation of individual parameters is mentioned in detail in Hebbar et al., (21). Here, we have used DC * Aggressiveness as the Pilot Inceptor workload (PIW) metric (29). Increase in PIW is a direct indicator of task difficulty.

## Results and Discussions

We used Pearson correlation coefficient to measure the association between pilot control behaviour-based PIW metric and physiological parameters. We generated one datapoint per user per condition where each row of physiological parameter came from one participant. Considering that each participant is a highly trained test pilot, sequence of task condition was regularly interchanged to avoid order effect and brief relaxation period was allowed between consecutive simulations; we assume independent observation across data points.

We also report repeated measures correlation (rmcorr) results for cases where strong association is observed with Pearson correlation. This is carried out to assess common intra-pilot association for the paired repeated measures data. rmcorr uses analysis of covariance to account for inter-individual variability (3). We used R Package (38) which is a statistical computing software to report the results. We report the magnitude and direction of both correlation coefficients in this section.

### Ocular parameter analysis

- Pupil dynamics analysis

**Figure 9.**
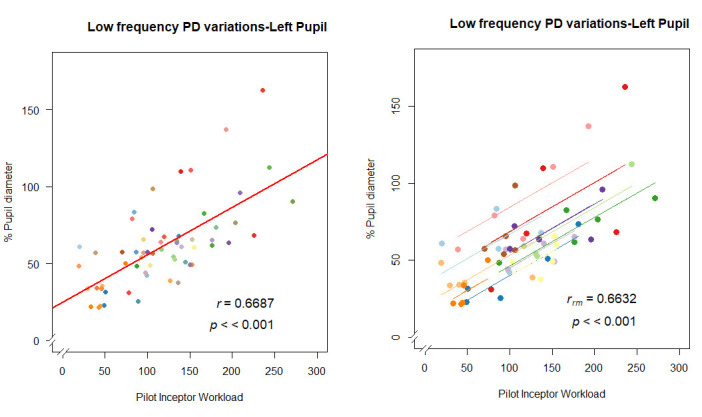

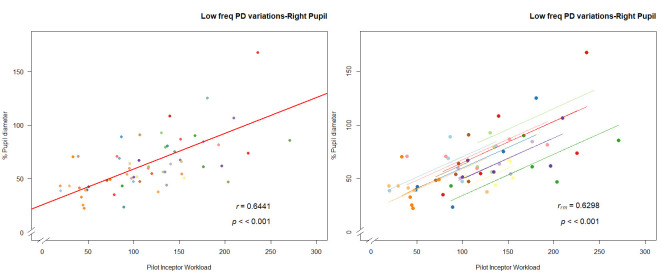
Correlation plot of low frequency PD variations: (a) left pupil- pearson correlation (left), rmcorr (right); (b) right pupil-pearson correlation (left), rmcorr (right).

We observed that low frequency PD variations show highly significant (p<0.001) strong positive correlation (r(58) = 0.668 for left pupil and r(58) = 0.64 for right pupil) with respect to PIW. rmcorr also confirms a positive relationship (r_rm_(47) = 0.66, p<0.001 for left pupil & r_rm_(47) = 0.63, p<0.001). Hence, we can infer that increase in the pupil diameter correlates with more abrupt pilot inceptor commands. (Figure 9).

b. Fixation analysis

**Figure 10.**
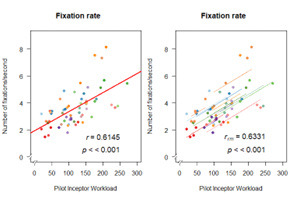

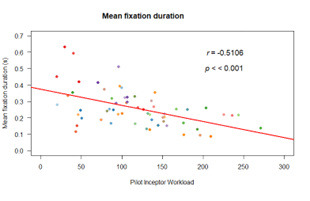
Correlation plot of Fixation analysis: (a) fixation rate - pearson correlation (left), rmcorr (right); (b) fixation duration

We observed significant (p<0.001) strong positive correlation (r(58) = 0.614, p < 0.001) between fixation rate and task difficulty and significant moderate negative correlation for mean fixation duration (r(58) = -0.51, p < 0.001) (Figure 10). rmcorr also computes a positive relationship for fixation rate with r_rm_(47) = 0.63, p<0.001.

c. Gaze distribution analysis

**Figure 11.**
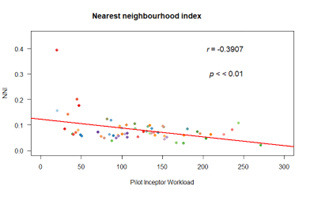
Correlation plot of NNI

A higher value of NNI denotes that visual scanning is more randomly distributed in space. We found moderate negative correlation between PIW and NNI (Figure 11) (r(58)=-0.39, p<0.01; (r(57)=-0.37, p<0.01 after removing the outlier of 0.4).

Decrease in NNI with task difficulty in VR is due to pilot’s increased focus and concentration on scanning of the target aircraft.

### EEG Analysis

**Figure 12.**
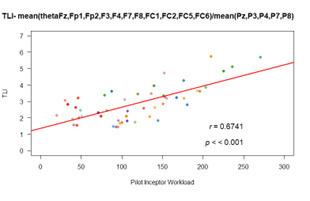

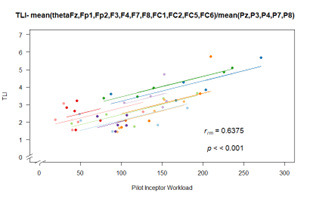
Correlation plot of TLI: (a) pearson correlation; (b) rmcorr (right)

- Task load Index:

We found that TLI shows highly significant (p<0.001) strong positive correlation (r(48)=0.67) with respect to PIW (Figure 12). rmcorr also confirms a positive relationship with r_rm_(39) = 0.64, p<0.001.

Hence increase in pilot’s activity increased the EEG task load index significantly.

b. Task engagement index

**Figure 13.**
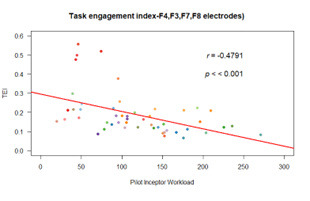
Correlation plot of TEI

We found highly significant (p<0.001) moderate negative correlation between TEI and PIW (r(48) = -0.48) (Figure 13).

A reduction of engagement index demonstrates the deterioration of task engagement with increase in pilot’s activity. We can further infer from the results that difficulty of the task consumed most of the attentional resources.

### Inter-pilot variability

Piloting skills and the perceived cognitive load varies with each pilot; for the same task condition. In this section, we present the results of analysis conducted to understand the inter-pilot variability across the estimated cognitive load parameters.

**Figure 14.**
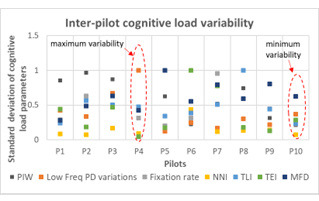
Inter-pilot variability

We computed standard deviation of the cognitive load parameters for the five task scenarios offered to each pilot. Figure 14 shows variability scores obtained across task conditions for each participant; for different cognitive load parameters. It can be observed that inter-pilot variability is relatively low for pilot 10 (represented by dark green dots in earlier figures). In other words, pilot 10 perceives more consistent cognitive load with respect to changes in task condition than others.

The overall observations from this study are:

- We measured pilot’s interaction with aircraft using his/her actions on the inceptor control through PIW metric. We further correlated PIW with ocular and EEG parameters-based metrics discussed in section 3.
- From the ocular parameter analysis, we found that variations in pupil dilation is an important cognitive load parameter. Strong position correlation is observed between increase in pupil diameter and PIW.

- We also noticed significant increase in fixation rate and reduction in length of fixation with increase in PIW. Results of the study corroborates the statement made in the previous section. The task reported in this article demands pilot to simultaneously process many flight parameters such as altitude, airspeed, orientation, distance from the target aircraft and number of remaining missiles to take decision to fire the missile. Decision-making process becomes more complex with increase in task difficulty; thereby resulting in an increased fixation rate.
- We observed that TLI shows strong positive correlation with increasing pilot activity. More importantly, we observed an inverse relation between TLI and TEI with increase in task difficulty. This negative association may be attributed to be due to nature of the task. TEI, by definition, explores information gathering, visual processing, vigilance and attention allocation. Decrease in TEI with PIW may be attributed to be due to decrease in availability of attentional resources and the amount of information processing due to pilot’s focus on tracking the target. This, in general, led to reduced peripheral activities such as visual scanning which is also evident from a reduced NNI with PIW (refer Figure 11).
- Initially, we assumed each data point as an independent observation and carried out Pearson correlation. These results were substantiated with repeated measure correlation. Table 2 shows the comparison between Pearson correlation coefficient (r) and repeated measure correlation (rmcorr) for all the cognitive load parameters reported in the paper. It can be observed from Table 2 that r and rmcorr values are comparable for parameters where strong correlation is observed.

**Table 2:** Comparison

Cognitive load parameter	Pearson correlation coefficient (r)	Repeated measure correlation coefficient (rmcorr)
Low frequency pupil dilation variations-left pupil	0.668	0.6632
Low frequency pupil dilation variations-right pupil	0.64	0.63
Fixation rate	0.614	0.6331
Mean fixation duration	-0.51	-0.53
NNI	-0.39	-0.142
EEG task load index	0.67	0.64
EEG task engagement index	-0.48	-0.37

Hence, in a VR environment, pupil diameter variations, gaze fixations, EEG task load index and EEG task engagement index are good indicators of cognitive load variations.

### Conclusions

We have presented the development of a virtual reality-based aircraft flight simulator that is proposed to be used to test new pilot vehicle interfaces. Realistic tracking scenarios are implemented. We conducted a user study with Air force test pilots to understand pilot’s interaction on the aircraft in an AI enabled battlefield scenario. No physical discomfort due to VR headset was reported during the conduct of simulations. Physiological measurement devices such as eye tracker and EEG headset are used for data collection. Physiological parameters were used to understand the variations in cognitive load during the pre-defined scenario simulations. Low frequency PD variations, gaze fixation rate and EEG task load index were found to be good indicators for estimating cognitive load in a virtual reality environment. We also observed that as pilot’s perceived task difficulty increased, more of pilot’s attentional resources were consumed. This was evident from both EEG based engagement index and from gaze fixation based NNI.

Analysis results indicate that the system estimates variations in the cognitive load efficiently. Hence, it is evident from the user study that VR flight simulator may be proposed for evaluations of new systems and their interactions in an iterative manner. Future studies should consider these results to evaluate new pilot friendly adaptive interface designs.

### Ethics and Conflict of Interest

The authors declare that the contents of the article are in agreement with the ethics described in http://biblio.unibe.ch/portale/elibrary/BOP/jemr/ethics.html and that there is no conflict of interest regarding the publication of this paper.

### Acknowledgements

Authors would like to thank all pilots who contributed for the data collection and provided very useful feedback for improving the task scenarios.
